# The Moderating Role of Pro-Innovative Leadership and Gender as an Enabler for Future Drone Transports in Healthcare Systems

**DOI:** 10.3390/ijerph18052637

**Published:** 2021-03-05

**Authors:** Hans E. Comtet, Karl-Arne Johannessen

**Affiliations:** 1 The Intervention Centre, Oslo University Hospital, 0372 Oslo, Norway; karajoha@online.no; 2 The Department of Design, Norwegian University of Science and Technology, 7491 Trondheim, Norway

**Keywords:** drones, unmanned aerial vehicle (UAV), healthcare, transport, socio-technical system, innovation

## Abstract

Drones have been suggested as an emerging technology that has the potential to disrupt and improve healthcare. The attitude among healthcare workers towards the use of drones is important for its successful implementation. Our aim was to examine individual and institutional variables among employees that may be relevant for the successful implementation of drones. This study used a self-administered questionnaire to investigate the expectations and beliefs among 400 employees at three Norwegian healthcare organizations regarding the future role of drones in the provision of healthcare. The results showed that the use of drones in healthcare is positively perceived across professional groups, age, and location. Working in an innovative environment, having experienced previous technological change in one’s working environment, and having leadership that supports new ideas were identified as drivers of individual beliefs regarding the use of drones as an innovative solution in future healthcare services. Men had significantly higher scores than women, and this was associated with reporting innovative leadership. This may indicate that a future implementation of drone usage should focus on local system environments and may depend on the presence of innovative leadership. Our results are harvested from a developed health care system and should be applicable for similar technologically advanced systems where the full potential of drone solutions may benefit from the integration of drones into the overall socio-technical system.

## 1. Introduction

Technological developments are enabling new solutions and challenging how we deliver existing services to an extent that was unimaginable only a few years ago [1]. Healthcare is an area that is facing large changes with respect to new treatment modalities, automatization, robotization, artificial intelligence, and innovations in existing methods aiming to individually tailor services and treatment. Technology has been suggested as one remedy that may alleviate the surging costs of healthcare. Demographic change, increased multimorbidity, and an increasing prevalence of lifestyle-related diseases in developed healthcare systems are all cost-driving factors [2]. It is also expected that technology may enable new solutions for the provision of services in less developed healthcare systems [3,4].

Unmanned aerial vehicles (hereafter referred to as drones) are one of several emerging technologies [5,6] that may contribute to this perspective. In healthcare, possible applications of drones have been investigated and include search-and-rescue operations in natural disasters, the delivery of vaccines and medicines to rural areas and healthcare technology devices in emergency situations, and the rapid transport of blood samples and organs [7,8,9,10,11,12,13,14].

Despite the challenges related to meteorological conditions, transport safety and security, and the short flight range, many researchers foresee drones as a transportation solution that could be viable within a few years [14,15,16,17,18]. The European Union expects that a fleet of 100,000 drones will be used for transport purposes by 2050 [19].

The institutional and clinical context in which drones may be a useful medium for the transport of biological material has not been extensively investigated, and whether the introduction of drones as a transport solution in healthcare will be a significant gain, remains unknown. Extreme demands for safety and uptime should be anticipated, and drones are still in an early innovation phase [20]. The speed of implementation as a transport solution may depend on differing sectors and purposes. Although multiple studies have demonstrated the proof of concept with respect to drone applications for multiple health care purposes [7,8,9,10,11,12,13,14], varying levels of momentum in the implementation of drones for high-volume and time-intensive transport in healthcare may be expected [8,21,22,23].

Furthermore, whether the implementation of drones for transport in healthcare systems will mainly be based on technical drone requirements alone or whether it will require specific organizational or system-related modifications may need further exploration [24]. As yet, the main focus of drones has been on their potential as airborne vehicles that can bypass ground-based traffic congestion [18] and provide accessibility for services in rural and remote locations [25,26]. It remains unclear whether such benefits and innovations can also be achieved for different transport options related to healthcare. This may concern organizational culture, logistics, and system improvements.

Although the implementation of drone solutions must be initiated and achieved at the local clinical or institutional level, an overall systemic perspective may contribute to understanding the scope of the necessary changes [3,27]. The impact of future regulations and the testing and use of drones [28], as well as interests from various stakeholders [29,30], may be relevant. The successful integration of drones may, therefore, benefit from a holistic socio-technical perspective that is adapted to healthcare cultures and systems [31,32].

The concept of “socio-technical systems” has been used to convey that the success of introducing new technologies does not depend only on the technical product itself, but also on overall policies, user experiences, infrastructure, and existing organizational structures [33]. The relevance of organizational structures and expectations related specifically to the use of drones in hospital systems has been studied by Krey et al. [34] and others [4,35,36,37]. These authors agree that this is an important issue requiring further investigation.

In particular, there is limited study on whether drone solutions can be achieved in existing logistics and organizational patterns that are found in specific settings, such as high-volume transport across large healthcare institutions or time-critical transport that requires an extremely high and regular uptime. Optimal service design often starts by including stakeholders to shape the best environment for new solutions to strengthen the innovation process [38,39,40]. In this context, the attitudes and culture among employees in hospitals that are planning to implement drones as a future transport option need to be accepting of this new technology.

This study focused on three Norwegian healthcare organizations that are building new hospitals by 2030 and are considering drone transport as a major part of their future transport logistics.

The largest hospital is Oslo University Hospital (OUS), consisting of four hospitals located throughout the Oslo area: The National Hospital (Rikshospitalet with local, regional, and national services), Ullevål University Hospital (local, regional, and national services), Radiumhospitalet (a specialized cancer hospital), and Aker University Hospital (a local, central hospital). In 2019, OUS had a total patient activity of 94,000 hospitalizations, 45,000 day care treatments, and 874,000 outpatient consultations. The hospital has 24,000 employees, and patient treatment takes place at more than 40 locations within a distance of 20 km. OUS is a large hospital by European standards, providing services that span from local hospital treatment to advanced specialized services and transplantations. The hospital has a structure that is well suited for drone transport across institutional locations.

The Central Hospital of Innlandet services a 52,072 km${}^{2}$ area (an area slightly larger than Denmark) and covers 42 local communities with a total of 368,000 inhabitants. The hospital has 8700 employees, with patient treatment taking place at 40 primary locations and 25 emergency locations. This organization is planning to have a centralized hospital structure by 2030, with one main central institution and three additional institutional locations. The hospital is currently exploring the use of drone services for conducting primary logistics across its institutional locations and between 80 local healthcare centers (i.e., general practitioners).

The Central Hospital of Vestre Viken is located west of Oslo and provides health services to 26 communities with 500,000 inhabitants. This hospital services a nearly 20,000 km${}^{2}$ area with a maximum distance of 180 km. The hospital has 9500 employees in total and institutions at five different locations. This organization is planning a new main hospital in the city of Drammen and is considering drone service as a future transport solution.

Our main goal in this study was to examine individual and institutional variables among employees that may be related to the expectations of and positivity towards future drone transport to identify the possible relevance of these variables for the successful implementation of drones.

## 2. Approach and Research Questions

We applied aspects from the multilevel perspective (MLP) developed by Geels et al. [41,42] related to socio-technical systems. This perspective investigates interlinkages between multiple aspects of an organizational system rather than as isolated phenomena in a local microsystem [43].

MLP distinguishes three levels: micro (niche), meso (regime), and macro (landscape), and these terms can parallel the institutional levels of clinic, hospital, and healthcare, respectively. MLP has the potential to increase our understanding of the dynamics that occur in the innovation phases of technological change in such systems [33,44].

MLP defines the technology itself as the core of the technologic change approach [42,45]. It conceptualizes four phases of technological change [44,46]: (1) Technology is introduced within the existing environment, (2) functionalities and user preferences are explored, (3) the change is put into practice in daily operations, and (4) gradual replacement of existing solutions.

These four phases describe the innovation journey that is characterized by various activities and dynamics. Niche innovations become gradually more specific and stable. The process of implementing niche innovations may benefit from positive feedback mechanisms that add new resources to the iterative development. However, if expectations are not met, then faith in the new technology might diminish and resources for it might be withdrawn [47].

Our current drone study was related to the first two phases. We used a structured questionnaire to obtain information related to organizational and technological topics among 400 employees from the three above-mentioned healthcare organizations.

## 3. Methods

A self-administered questionnaire containing 36 structured questions (Appendix A) was conveyed by mail, published on the organization’s intranet, or presented to randomly contacted employees during workdays (in the case of OUS and Vestre Viken). We received a total of 415 responses: 39 by mail, 234 by intranet, and 142 by random contact. The responders were categorized into professional groups as presented in Table 1.

We chose random sampling for a study group of approximately 400 subjects. Although a representative sample should contain, for example, 80 physicians (based upon the personnel structure in the hospitals), selection of 80 physicians across 45 differing specialties in the hospitals might represent a selection bias using representative sampling, as such a method would need a weighted sampling across small and large specialties. Furthermore, whereas representative sampling minimizes bias from known causes, random samples minimize bias from unknown causes [48]. We had no control of the respondents on the intranet questionnaires, and therefore chose a random sampling method for all questionnaires. This resulted in a fairly representative study group, as the percent of the differing personnel categories in our sample versus relative number of workforce were administration 15%/14%, bioengineer 14%/8%, nurse 31%/39%, other 12%/12%, other patient 14%/10%, and physician 19%/17%.

We had three targets of information in our survey. We aimed at capturing “the overall picture” among healthcare employees regarding the individual digital interest and competence, knowledge, and expectations of future drones, some indicators of the local cultures. Multiple variables might be used as we had no prior knowledge of the status. We also intended to limit the time to perform the response, anticipating that much time for filling in the questionnaire could be negative. The 36 questions consisted of 16 yes–no questions, 13 multiple-choice questions, and a Likert scale (rating of 1–5) questions. We tested the questionnaires before the study using three physicians, three nurses, and two secretaries to assess the time needed to fulfill the questionnaire and solve ambiguous questions.

Information of our study was presented on the intranet of the three hospitals. The survey questions alternated between yes/no, multiple-choice, and Likert-scale response options, intending to make it more interesting and appealing to answer [49]. For the survey that was distributed physically to employees at the hospitals, we addressed the employees by inviting them to participate for an important health-related research, i.e., we asked as insiders. Of course, avoiding some respondents that intentionally reply with wrong answers might be difficult to control for in survey-based studies.

The questions collected demographic and background data of the responders, their knowledge of drones, experience with technological change, and attitudes/expectations for the future. The demographic characteristics included profession, gender, age, self-assessed digital competence, currently working under innovative leaders, and working in a culture resistant to change. The technological perspective of the responders was inquired by asking about their knowledge of drones in general and in healthcare, personal beliefs regarding drones in the future, experience of radical technological change (not specified), and anticipated digital implications for their career. Attitudes/expectations for the future were gathered by asking about their expectations of the new hospital, expectations of upcoming technologies for improving healthcare, and perceived threats to their own career development posed by technological change.

We excluded 15 responses because of missing data. The questionnaire data were transferred into Excel for descriptive analysis. The statistical analyses were performed with SPSS version 27. Differences between groups were examined by two-way analysis of variance, and significant Pearson correlations between single variables were used to perform multivariate analyses. To assess whether the employees believed that drones will be implemented in future healthcare services (yes/no), we used stepwise logistic regression with this dichotomous variable against multiple variables. We used dummy variables for personnel groups and the institution. A grouping variable of gender (man = 0, woman = 1) was applied to assess the differences between men and women. A significance level of *p* < 0.05 was considered to be statistically significant.

## 4. Results

A summary of the core variable data is presented according to profession in Table 1. There was a majority of women responders (64%), but the physician group had a significantly higher percentage of men (57%) than women. The mean age was lowest in the nurse group (*p* < 0.01 compared with the total sample).

Administrative staff, nurses, and physicians had the highest belief that in the future drones would be used in healthcare (*p* < 0.05); however, there was no such trend related to future drone transport at their own hospital. The personnel groups that are closest to patients, i.e., nurses, other patient-related personnel, and physicians, had the lowest expectations of a new, future hospital with respect to improvements in the patient and employee perspective. Administrative personnel scored the lowest on having experienced radical change, while bioengineers scored significantly higher on this topic (*p* < 0.01).

There were some score differences across gender (Table 2). Significantly more men had knowledge of drones in general and an awareness of drones in healthcare; however, the belief that drones would be used in future healthcare services was similar between men and women. The scores of women reflected more skepticism regarding the future risk of general technological development in healthcare.

**Figure 1:**
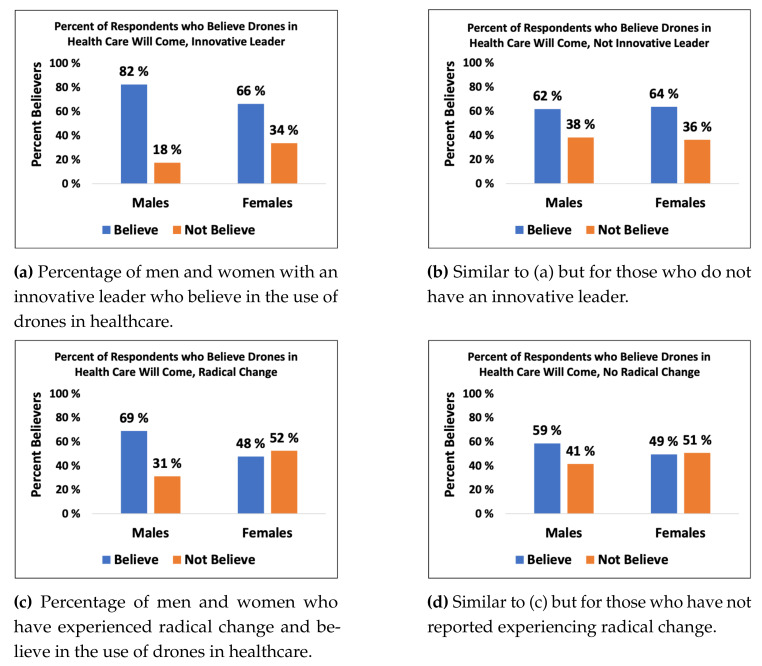
Interesting covariations that show (**a**) percentage of men and women with an innovative leader who believe in the use of drones in healthcare, (**b**) percentage of men and women without an innovative leader who believe in the use of drones in healthcare, (**c**) percentage of men and women who have experienced radical change and believe in the use of drones in healthcare, (**d**) percentage of men and women who have not experienced radical change and believe in the use of drones in healthcare.

Several variables had interesting covariations. Having innovative leadership increased one’s belief that drones would be used in future healthcare services. This was primarily seen in men, as 82% of men who worked in innovative cultures believed in the use of drones compared with 66% of women (*p* < 0.01). Similarly, for those who experienced radical technological change in one’s own work, a significantly higher percentage of men (69%) than women (48%) (*p* = 0.015) believed that drones would be used in healthcare (Figure 1a–d).

A similar but weaker effect was observed in relation to having an arena for innovative communication; in this setting, 80% of men believed that drones would be used in future healthcare compared with 67% of women (*p* < 0.05).

Variables that had significant Pearson correlations were included in a multivariate logistic regression analysis using the belief in drone usage in future healthcare as a dichotomous dependent variable (1: believe, 0: do not believe). The results of the final step in a backward analysis are presented in Table 3.

Regarding profession, physicians and administrative personnel showed the largest odds ratio for the belief in drone usage in future healthcare, and experience of radical technological change and having an innovative leader were the strongest environmental predictors. Interestingly, increasing age was associated with a lower optimism for the use of drones in healthcare.

## 5. Discussion

Our results showed that an average of 70% of the participants envisioned drones as a realistic transport solution in future healthcare. This finding was similar across multiple personnel groups, although administrative employees, physicians, and nurses had the highest belief in drone usage. Interestingly, the idea that their own hospital would implement drone solutions was somewhat lower.

The high trust in drones as a future transport solution in health services may be interesting in several aspects. For such subarctic climatic conditions as in Norway, with temperatures varying from −30 to +30 degrees Celsius and wide ranges in wind and precipitation, it has not been proven that the current drone technology may provide near 100% uptime with acceptable quality of biologic specimens. Although a recent study reported that both normal and pathologic blood samples may tolerate exposure to vibration and turbulence [23], multiple challenges, such as icing of drone propellers and wings, and drone range and turbulence around buildings and infrastructures, remain to be studied. We do not believe that our study group has an informed envision based on such topics for their positive assumptions of future drone solutions.

On the other hand, we may interpret our findings as our health care workers have high fidelity in upcoming technologies, that they have heard about and observed drones in general, and therefore have an enthusiastic expectation to drones in the future.

With a socio-technical approach based on individual reported profiles, our study intended to identify possible topics related to technology and local variables in existing systems that may be associated with drone implementation.

Our results demonstrate positive attitudes toward drones among healthcare workers across multiple professions. These workers reported a mean self-assessed digital competence score varying from 3.6 to 3.9 on a 1–5 Likert scale. They had a reasonably high knowledge of drone usage in general and in healthcare, high confidence that technological solutions may improve health services in the future, and high expectations for technology to improve their own work. The employees’ concern for their jobs related to future technological change was low across all groups. These observations may, in part, reflect the positive attitude towards technology displayed by the Norwegian population [50].

Experiencing innovation in the local environment, having an innovative leader, and working in an arena that simulated innovation were all positively correlated to the belief in using drone solutions in future healthcare. A similar effect was observed for experiencing radical technology changes in the course of work. However, we cannot conclude whether such individual responses represent local working environments or whether they merely represent individual attitudes. Nevertheless, the significance of such factors with respect to the implementation of drone transport in future health systems should not be ignored, as previous reports have suggested that these topics may be relevant if implementing specific technologies can generate a broader acceptance of general technological innovation [1,3,24]. Our results are in accordance with previous studies that have suggested that greater experience leads to increased familiarity and the ability to learn about technology in general [51] and that the experience and knowledge of early adopters/believers can positively contribute to further technology implementation [52].

The full impact of the gender difference in the current study on the introduction of drones in healthcare is not known. Notably, the effect of working in an innovative culture was a strong factor for men but less for women (Table 2). In contrast, the response profile was quite similar for the two genders in the group that did not report innovative leadership. Whether this may indicate that men are more receptive to innovative cultures than women would need more research. Gender differences related to drones was discussed in a previous report of food drone delivery by Hwang et al. [53]. They found that women who perceived a technology to be innovative were more likely to use the technology than those who did not perceive it to be innovative.

In Norwegian hospitals, 65% of employees are female. With a managerial lens, any gender difference may be of importance related to organizational and innovative developments. If drones are emerging as realistic solutions for the purpose that our hospitals are aiming, understanding possible differences across personnel groups and genders may be useful for the implementation of drones in an extended framework. If we assume that women have a less receptive attitude to innovative cultures than their male colleagues, this should be understood and taken into account.

In our study, administrative personnel, physicians, and nurses showed the largest odds ratios for believing that drones would be used in future healthcare, although they did not stand out with significant higher scores on either digital competence or knowledge of drones. One hypothesis may be that having a higher academic education may be associated with more engagement in future technology. The high score in the administrative group may also be associated with open minded attitude related to managerial positions, although we have no detailed information on their background.

As nurses and physicians are professions close to the clinical activities, they may have more positive experience with the continuous changes in technology and patient treatment over time, although not all changes are observed as radical. As they represent influence on the provision of clinical services, they potentially may be advocates for the introduction of drone solutions, if such solutions are concluded realistic in the future. Further research is needed to fully understand these findings. In a similar study of the Swiss system, Krey et al. [34] reported different attitudes to drones between employees and patients and between healthcare-related personnel and other employee groups. Our results are different from those of Krey et al. because the administrative group had the highest score in our study. This indicates that such factors may vary across different systems and cultures.

The results in Table 3 illustrate several interesting odds ratios Exp(B). The odds ratio for believing in future drone solutions had the largest value in administrative personnel and physicians (2.73 and 2.43, respectively). This is 89.5% and 68% higher than the odds ratio of the general knowledge of drones, and 52.5% and 37% higher than the odds ratio of knowledge of drones in health care. In comparison, the odds ratio of physicians deviated only 25% from that of nurses. These numbers may spark several research questions regarding possible causes as academic background, professional attitudes and cultures, experience from technology, and more.

People’s knowledge of emerging technologies may be incomplete and based on their personal values and knowledge constructs [54]. A lack of involvement and knowledge might generate skepticism, whereas positive experiences of radical technological change may build attitudes that are more accepting of change. This idea is consistent with our finding that previous experience with radical change supported a positive attitude toward the use of drones in future healthcare.

Both Mion [36] and Knoblauch et al. [4] discussed the importance of having dedicated project management to support the implementation of drones through the various innovation phases. Change is not driven by the technological solution alone but in combination with the adoption by users, which helps structure the solutions [33,55]. Incentives for experimentation, learning, network building, and vision building have been suggested as crucial elements in the push for change [44,52,56]. These ideas are universal principles, and they correspond to our findings that innovative and supportive management strengthens the belief in future drone solutions.

Negative experiences with a new technology may create acceptance problems whereby people become resistant to subsequent changes [44,57,58]. By contrast, positive experiences and anecdotes may enhance the cultural appeal and social acceptance at the local level [44,52,56]. Users may gradually learn through practical interactions with technology and explore new ways to organize workflows [45].

We found that our study group was receptive to new solutions; however, differing institutional and individual attitudes should be considered by the local leadership and management in future drone implementations. A socio-technical approach might be helpful in this implementation process, as it highlights the importance of using technological transformation as an opportunity to improve processes by combining organizational and technological needs [59,60].

The ability to change and improve existing solutions by implementing technological innovations—in contrast to modifying new solutions to fit existing routines—may become a critical factor for the success of future healthcare organizations.

Drone transport may have the potential to reduce costs by merger of large laboratory services that are traditionally performed at multiple locations with the duplication of infrastructure and 24/7 services. If drones represent a transport system that offers close to 100% uptime with sufficient quality, they may contribute to the centralization of time-critical laboratory services, reducing both operational costs and costs of infrastructure investment. Additionally, drones may also represent decentralization by increasing the pick-up services in rural regions by more frequent and timely transport schedules than current road transport. This may offer both reduced costs for the health care and improved quality for patients.

The full potential of implementing such solutions into existing health care systems may depend on logistics and cooperation across institutional and district systems, thus asking for an extended, socio-technical framework [24,59]. Exploring and understanding the readiness of an organization may therefore be a crucial factor for the implementation of drones. Mion [36] emphasizes that organizations intending to use drones for transport need to be open for change and prepared to modify their operations. Although focusing on the microlevel may be crucial in the initial implementation phases, a systemic perspective may facilitate integrating drones into the healthcare system by considering the entire process, encompassing both social and technical aspects. This would help to improve current work practices and realize the full potential of drones in the long run. Socio-technical systems do not work on their own, but in an interplay between technology and the user environment [42]. Innovative leadership and culture may strengthen the acceptance and implementation of using drone transport in healthcare. Furthermore, it may shape the organizational structure that may be necessary to adapt to technological change.

If drones are emerging as realistic solutions for the involved hospitals, their implementation should attempt to obtain improvements of processes and cultures beyond the primary transport topic. The practical implications of our results may therefore be multiple. The conceptual framework of attitude and knowledge among health care workers defines the framework for decision-makers of how management and leadership should be performed to attain a disrupting technology adoption in an extended healthcare logistic process. Observing that there may be differing attitudes across the professions and possibly across genders, may be of significant importance.

Our data are harvested from hospitals that have declared drones as a future transport strategy. This may imply differing attitudes compared to other systems. Our findings may therefore be influenced by such specific factors. However, we observe hospitals in several health care systems are developing such strategies and give some generality for our results.

No doubt, implementations of drone solutions in less developed systems may have quite differing challenges as those in more developed systems. In our context, all three hospitals intend improve costs by merging large laboratories, but in combination with improvements in decentralized services, as drones may increase service level in rural and remote locations. The latter parallels demand in many lesser health care systems as well. Nevertheless, observing differing attitudes and expectations across employee groups will have general validity. In particular, in a socio-technical analysis, we conclude that gender, innovative leadership, and innovative arenas may be relevant elements to study when implementing any technological innovation.

## 6. Strengths and Limitations

Our study is subject to some limitations. The findings are limited to a Norwegian setting. Norway is a high-expender, ranked among Organization for Economic Co-operation and Development countries (OECD) from second to fourth in terms of total health expenditure per capita [61]. This may influence attitudes related to economic expectations for new solutions. Furthermore, Norway, along with other Scandinavian countries, is a leader regarding equity policies. Gender differences may diverse from other health care systems.

We did not define the term “drone” but assumed that most Norwegians associate the term drone with a standard multicopter drone which is regularly shown in the media and is owned by half a million Norwegians.

Although this study included more individuals than in similar previous studies and our results may be valid at the individual level, a limitation of our study is related to survivorship bias [62]. Recruiting respondents via the organization’s intranet may have biased the responders towards those who have a special interest in the topic, and they may not be representative of the overall employee population. This may have caused some selection bias in our study group, and we cannot make conclusions on a local clinic or institutional level. Nevertheless, the study group provided interesting perspectives at the individual level across fairly broad personnel groups.

Using a self-administered questionnaire may have introduced errors related to different understandings and interpretations of the questions. Survey questionnaires do not allow for follow-up of the answers and are not suited for a deeper investigation of the responses [63]. Furthermore, our study was carried out in a technologically advanced healthcare system, and the findings may not be as relevant to less developed systems [34]. However, the association between an innovative culture and the successful innovation and implementation of technology has been suggested by others [64] and is supported by our findings.

## 7. Conclusions

This study suggests that drones are perceived positively across a variety of professions, ages, and locations. A major finding was that working in an innovative environment, having a leader that supports new ideas, and having previous experiences with technological change are drivers for the belief that drones will be used in healthcare and the positive perception of future technology transitions. Our findings require further research related to the impact of the investigated factors on the establishment of drone solutions. However, we believe that to realize the optimal and extensive benefits of implementing drone solutions, the managerial approach should be open minded, stimulate innovation, encourage experimentation, and support the risk-taking that will be necessary.

## Figures and Tables

**Table 1:** Summary of data by personnel group.

Profession	Administration (*n* = 59)		Bioengineer (*n* = 54)		Nurse (*n* = 110)		Other (*n* = 47)		Other Patient Related (*n* = 55)		Physician (*n* = 75)		Total Population	
**Category/Variable**	**Mean**	***SD***	**Mean**	***SD***	**Mean**	***SD***	**Mean**	***SD***	**Mean**	***SD***	**Mean**	***SD***	**Mean**	***SD***
Background														
Mean Age (years)	41.2	11.5	39.8	12.4	32.9	12.1	39.1	13.1	39.0	11.4	35.2	12.9	37.30	14.3
Years Worked in Hospital (years)	13.5	9.5	14.8	12.4	9.3	10.2	10.1	10.7	9.3	9.9	7.2	8.3	10.6	9.6
Self Assessed Digital Competence (score 1–5)	3.8	0.7	3.9	0.8	3.7	0.7	3.8	0.9	3.8	0.9	3.6	0.9	3.8	0.64
Positive Culture for Change (% yes)	88%	33%	74%	44%	90%	30%	78%	42%	73%	45%	83%	38%	82%	38%
Innovative Leadership (% yes)	83%	38%	74%	44%	84%	37%	70%	47%	71%	46%	69%	46%	76%	43%
Arena for Innovation (yes)	59%	50%	59%	50%	62%	49%	65%	48%	49%	50%	55%	50%	57%	50%
Knowledge of Drones														
Knowledge of Drones in Health Care (% yes)	73%	45%	76%	43%	56%	70%	72%	62%	55%	63%	49%	62%	62%	63%
General Knowledge of Drones (score 1–5)	2.93	0.92	2.75	0.97	2.32	0.82	3.0	1.20	2.55	1.12	2.64	1.13	2.7	1.0
Believe Drones in Future Health Care (% yes)	81%	39%	67%	48%	73%	45%	54%	50%	60%	49%	77%	42%	70%	45%
Believe Drones in Own Hospital in Future (% yes)	66%	48%	63%	49%	67%	47%	74%	44%	53%	50%	64%	48%	65%	48%
Technological Experience and Expectations														
Experienced Radical Technological Changes (% yes)	32%	47%	59%	50%	39%	49%	33%	47%	47%	50%	49%	50%	43%	49%
Believe New Hospital Positive for Employees (% yes)	68%	47%	65%	48%	66%	55%	70%	47%	64%	73%	53%	55%	57%	49%
Believe New Hospital Positive for Patients (% yes)	75%	44%	69%	47%	69%	52%	76%	48%	58%	66%	56%	55%	61%	48%
Believe Digitalization may improve Health Care (% yes)	97%	18%	98%	14%	98%	19%	98%	15%	98%	13%	99%	12%	98%	15%
Hospital need Change to adapt to Technol. Development (% yes)	92%	28%	87%	34%	83%	38%	65%	48%	80%	40%	75%	44%	82%	38%
Worried Own Work May be removed by Future Technology (%)	16%	39%	9%	32%	29%	39%	19%	46%	12%	37%	16%	36%	17%	37%
Positive Expectations Technology Improve Own Work (scale 1–5)	3.90	0.90	3.87	0.99	3.93	0.82	3.87	1.26	3.62	1.03	3.67	0.93	3.83	0.81

**Table 2:** Summary of data by gender.

	Males (*n* = 138)		Females (*n* = 262)					
Category/Variable	Mean	*SD*	Mean	*SD*	Mean Male/Female	Sig. (2-Tailed)	Mean Difference	Std. Error Difference
Background								
Mean Age	40.07	11.68	35.46	12.70	13%	0.01	3.25	1.87
Years Worked in Hospital	10.06	9.72	10.54	10.73	−5%	ns	−0.99	ns
Self Assessed Digital Competence	3.92	0.84	3.67	0.78	7%	0.01	0.25	ns
Positive Culture for Change	82%	39%	83%	38%	−1%	0.05	0.06	<0.05
Innovative Leadership	75%	44%	77%	42%	−3%	ns	−0.02	<0.05
Arena for Innovation	56%	50%	60%	49%	−7%	ns	−0.03	<0.05
Knowledge of Drones								
General Knowledge of Drone	3.08	0.97	2.40	0.99	28%	0.001	0.69	<0.01
Knowledge of Drones in Health Care	67%	51%	59%	65%	15%	0.00	0.20	<0.05
Believe Drones in Future Health Care	78%	42%	66%	47%	17%	0.01	0.02	<0.05
Technological Experience and Expectations								
Experienced Radical Technological Changes	43%	50%	43%	50%	1%	ns	−0.00	ns
Believe New Hospital Positive for Employees	64%	55%	64%	55%	1%	ns	0.04	ns
Believe New Hospital Positive for Patients	67%	52%	67%	54%	−1%	ns	0.03	ns
Believe Digitalization may improve Healthcare	95%	22%	99%	9%	−4%	ns	−0.04	0.02
Hospital need to Change to adapt to Technological Change	1.28	0.36	1.41	0.41	−9%	0.04	−0.18	ns
Worried Own Work May be removed by Future Technology	14%	34%	19%	39%	−26%	0.01	0.01	<0.05
Positive Expectations for own Work	3.80	0.87	3.82	1.02	−1%	ns	−0.08	ns

ns = not significant.

**Table 3:** Multivariate logistic regression with a belief in drone usage in future healthcare as the dichotomous dependent variable.

Variable	B	S.E.	Wald	Sig.	Exp(B)
General Knowledge of Drones	0.36	0.14	6.46	0.001	1.44
Knowledge of Drones in Healthcare	0.57	0.22	7.08	0.008	1.773
Experienced Radical Technological Changes	0.66	0.26	6.61	0.010	1.929
Innovative Leadership	0.63	0.31	4.18	0.041	1.872
Believe Digitalization may improve Healthcare	0.60	0.14	18.32	0.000	1.825
Nurse	0.67	0.31	4.69	0.030	1.95
Physician	0.89	0.36	5.97	0.015	2.43
Administration	1.00	0.40	6.32	0.012	2.73
Age	−0.02	0.01	4.27	0.039	0.98
Constant	−1.10	0.98	1.27	0.260	0.33

B = Coefficient (intercept); S.E. = Standard error; Wald = Wald chi-square test; Sig. = *p*-value; Exp(B) = Odds ratio.

## Appendix A

### Questionnaire

- Your profession? *Physician, Nurse, Bioengineer/laboratory employee, Other patient-related work, Administration, Other*
- How do you rate your digital competence? *Manage—Superuser*
- Do you think digital solutions contribute to more efficient health services? *Yes/No*
- Do you think a new hospital will be beneficial for employees? *Yes/No*
- Do you think a new hospital will be beneficial for patients? *Yes/No*
- What do you know about drones in general? *Very little—Very much*
- Have you heard about drones in healthcare? *Yes/No*
- Do you think a future drone-based transportation of biological material (blood samples, biopsies, other material) is realistic? *Yes/No/Don’t know*
- In which area do you think a drone-based transportation can have a positive effect? *Time, Quality, Digital, Collaboration and Communication, Don’t know*
- In which situations do you think a drone-based transportation can be an advantage over current transportation methods? *Long distances, Predictability, Possibility for immediate transport, Consolidations of laboratories, Don’t know*
- Do you think your hospital will use drones in the future? *Yes/No/Don’t know*
- How long do you think it takes to fly a blood sample taken at Rikshospitalet with a drone to Ullevål? *0–15, 15–30, 30–45, 45–60, 60–90 min, Don’t know*
- What do you believe are the biggest risks when it comes to drone flights for medical purposes? *Flight safety, Data safety, Biological quality, Sample safety*
- Does your leader support innovative ideas? *Yes/No*
- How does your leader react to innovative ideas? *With rejecting/With doubt/Open/With interest/With support*
- Do you have an arena to discuss and/or test innovative ideas in your unit? *Yes/No*
- Are you leaders active in planning for future change? *Yes/No*
- Are you involved in the planning for future change? *Yes/No*
- Do you think that technological development poses a risk for your hospital? *Yes/No/ Don’t know*
- From your perspective, does technological development require that the hospital needs to change? *Yes/No/Don’t know*
- Are you optimistic that technology can improve your work in the future? *Pessimistic—Optimistic*
- Are you concerned that your work might disappear or that you might lose work as a result of technological development? *Yes/No*
- Have you experienced significant medical-technical change in your area the last 5–10 years? *Yes, radical/Yes, to some degree/Some, but less significant/No*
- If you answered yes to the previous question, did technological development lead to logistical/operational change? *Not relevant/Yes/No*
- How satisfied are you with your work today? *Not satisfied—Very satisfied*
- What gives you most work satisfaction? *Interesting work/Work with patients/Work autonomy/No work-related stress/Nice colleagues/Exciting change projects/Busy and the day goes fast/Good at my work/New challenges*
- Do you experience good collaboration and communication with other professions in your work? *Disagree—Agree*
- Do you have enough time during the day to accomplish your work in a satisfactory way? *Yes/No*
- Will your work be less or more interesting in the future? *Less interesting— More interesting*
- How important is it for you to know what you need to do during the day? *Not important—Very important*
- How often do you have to do unpleasant work? *Never/Rarely/Sometimes/Often*
- What are the reasons for that feeling? *Always behind/Poorly handled organisational change/Delayed work/Too much to do/Poor communication/No support/Inconsistent expectations/Too many patients/Concerned for patients*
- Do you think the hospital needs other then medical competence in the future? *Yes/No*
- What are the biggest challenges for the healthcare system from your perspective? *Shortage of resources/Increasing costs/Increasing unnecessary treatments/Competence/Poor communication/Specialisation*
- Your age? *0–19/20–29/30–39/40–49/50–59/60 years or older*
- Your gender? *Female/Male*
